# Artificial intelligence-based analysis and diagnosis of intradural extramedullary spinal tumors by stimulated Raman histology

**DOI:** 10.1093/noajnl/vdaf211

**Published:** 2025-10-08

**Authors:** Pierre Scheffler, Nicolas Neidert, Jakob Straehle, Daniel Erny, Marco Prinz, Ulrich Hubbe, Roland Rölz, Dieter Henrik Heiland, Jürgen Beck, Amir El Rahal

**Affiliations:** Department of Neurosurgery, Medical Center, University of Freiburg, Freiburg, Germany; Center for Advanced Surgical Tissue analysis (CAST), University of Freiburg, Freiburg, Germany; Department of Neurosurgery, Medical Center, University of Freiburg, Freiburg, Germany; Center for Advanced Surgical Tissue analysis (CAST), University of Freiburg, Freiburg, Germany; German Cancer Consortium (DKTK), Partner Site Freiburg, Freiburg, Germany; Department of Neurosurgery, Medical Center, University of Freiburg, Freiburg, Germany; Center for Advanced Surgical Tissue analysis (CAST), University of Freiburg, Freiburg, Germany; Institute of Neuropathology, Faculty of Medicine, University of Freiburg, Freiburg, Germany; Institute of Neuropathology, Faculty of Medicine, University of Freiburg, Freiburg, Germany; Department of Neurosurgery, Medical Center, University of Freiburg, Freiburg, Germany; Department of Neurosurgery, Medical Center, University of Freiburg, Freiburg, Germany; Center for Advanced Surgical Tissue analysis (CAST), University of Freiburg, Freiburg, Germany; Department of Neurosurgery, Medical Center, University of Freiburg, Freiburg, Germany; Center for Advanced Surgical Tissue analysis (CAST), University of Freiburg, Freiburg, Germany; Department of Neurosurgery, University Hospital Erlangen, Erlangen, Germany; Microenvironment and Immunology Research Laboratory, Friedrich-Alexander University of Nürnberg-Erlangen, Erlangen, Germany; Translational Neurosurgery, Friedrich-Alexander University of Nürnberg-Erlangen, Erlangen, Germany; Department of Neurological Surgery, Northwestern University Feinberg School of Medicine, Chicago, Illinois (D.H.H.); Department of Neurosurgery, Medical Center, University of Freiburg, Freiburg, Germany; Center for Advanced Surgical Tissue analysis (CAST), University of Freiburg, Freiburg, Germany; Department of Neurosurgery, Medical Center, University of Freiburg, Freiburg, Germany; ; Faculty of Medicine, University of Geneva, Geneva, Switzerland

**Keywords:** artificial intelligence, intradural extramedullary tumors, stimulated raman histology

## Abstract

**Background:**

Intraoperative Stimulated Raman Histology (SRH) has been reported to be fast and accurate in the assessment of neuro-oncological lesions. However, its application to spinal tumors, especially intradural extramedullary tumors (IDEM), remains underexplored. IDEM primarily include meningiomas and schwannomas, as well as less common entities such as metastases or ependymomas. Given that surgical resection is the primary treatment modality, rapid, artificial intelligence (AI)-driven intraoperative tumor classification based on SRH could enhance surgical decision-making and subsequent management.

**Methods:**

We acquired 232 SRH images from patients with IDEM using the NIO Laser Imaging System (Invenio Imaging Inc.). We categorized images into three diagnostic classes: “Meningioma,” “Schwannoma,” and “Other.” Images were divided into 224 × 224 pixel patches and used to train and test AI-based image classifiers employing CTransPath, ResNet, and Vision Transformer architectures.

**Results:**

Our best-performing model, utilizing the CTransPath architecture, achieved a classification accuracy of 94.3% on the test dataset. Vision Transformer-based models also performed well, exceeding 90% accuracy, while ResNet models attained slightly lower accuracies (79.6%-88.8%). Qualitative analysis indicates that the top-performing model primarily relies on cellular morphology for classification.

**Conclusions:**

Our findings confirm the feasibility and effectiveness of AI-assisted SRH analysis for distinguishing IDEM tumor types. This approach may complement conventional intraoperative neuropathology by providing rapid, reliable, and clinically actionable diagnostic information.

Key Points

- AI can distinguish most IDEM tumors in SRH
- This enables near real-time intraoperative histology

Importance of the Study

Intraoperative diagnosis of IDEM currently depends on frozen sections, which are accurate but resource-intensive. Although SRH is established in brain tumors, its use for spinal IDEM and AI-assisted classification has been scarcely explored. We present, to our knowledge, the first deep-learning approach tailored to SRH-based identification of IDEM, reaching 94.3% accuracy with a histopathology-pretrained hybrid architecture. Delivered within minutes and without manual interpretation, AI-interpreted SRH can complement conventional intraoperative pathology. Clinically, rapid differentiation of meningioma versus schwannoma may guide resection strategy, especially regarding nerve-root preservation.

Intradural extramedullary tumors (IDEM) are tumors that arise inside the dura mater but outside the spinal cord parenchyma. They are the most common type of intradural spinal tumors, a rare category of tumors with an incidence of 0.3 per 100.000 per year.^1^

Most IDEM are benign lesions that can lead to neurological deficits through compression of the spinal cord, cauda equina, or spinal roots. The majority of IDEM are meningiomas and schwannomas.^1^ Whereas meningiomas arise from cells of the arachnoid, spinal schwannomas originate from Schwann cells of spinal nerve roots. Surgery is currently considered the primary treatment strategy for both entities.^2^ Although lesions are ideally resected completely, this is associated with a risk of permanent neurological deficits, especially in the case of schwannomas due to their association with spinal nerve roots. Pre- or intraoperative identification of tumor entities would allow for improved surgical decision-making; however, preoperative MRI imaging is often unable to distinguish most IDEM, with most tumors presenting as homogenously enhancing masses.^3^

Stimulated Raman Scattering Histology (SRH) is a novel histopathological imaging technique that enables label-free chemical contrast within minutes.^4^ It is based on the Raman effect, the inelastic scattering of incident photons. SRH can be used for intraoperative histopathological diagnosis with imaging quality comparable to hematoxylin and eosin (H&E) sections. Previous publications have shown that SRH and conventional histopathology have similar diagnostic accuracy, and it can easily be integrated into the workflow of neurosurgical tumor resection.^5^^,^^6^ SRH can even be used to reliably predict molecular features such as IDH mutations and 1p19q codeletions and to quantify the degree of tumor infiltration in gliomas.^7^^,^^8^

As SRH creates digital histopathological images in a consistent and reproducible manner, SRH images represent an excellent dataset for training neural networks. In the past decade, computer vision has made significant progress, with some neural network architectures, such as Vision Transformers (ViT) and convolutional neural networks (CNN), achieving human-level performance in image classification tasks if given adequate training data.

CNNs are the current standard for deep learning-based computer vision models. They rely on convolution operations between successive layers of neurons, in which every neuron receives input from a restricted area of the previous layer, similar to the receptive field of neurons in the visual system of animals. One of the most widely used CNN models is ResNet.^9^ ResNet is a deep neural network architecture based on numerous consecutive convolution operations that can be intermittently skipped with residual connections; this makes it possible to train much deeper networks effectively. ResNet represented the state-of-the-art neural network architecture for image classification tasks upon its release and is still frequently used for such tasks due to its relatively small size and robustness during the training process. This CNN has already been successfully used in earlier studies for medical image classification tasks such as tumor segmentation and classification of MRI or intraoperative images.^10^^,^^11^

Recently, vision transformers have become a competitive alternative to convolutional neural networks in imaging tasks. Vision Transformers are based on the original transformer architecture, as published by Vaswani et al,^12^ that uses the attention mechanism to learn complex relationships between tokens. Although these models were originally used for language translation, Dosovitskyi et al^13^ showed that the concept could be extended to computer vision by splitting images into fixed-size patches that serve as tokens, demonstrating impressive results in image classification, particularly when trained on large datasets. Its accuracy was found to be comparable to the ResNet architecture in a recent publication in histopathological analysis.^14^

In our study, we investigated whether different neural network architectures based on either a convolutional neural network, a vision transformer, or a hybrid architecture could be used for automated analysis of SRH images to differentiate between intradural extramedullary tumors such as schwannomas, meningiomas, and other spinal tumors.

## Materials and Methods

### Sample Collection

Samples were collected intraoperatively from patients undergoing surgery for suspected intradural extramedullary tumors. The local ethics committee approved sample collection and analysis (application number 23-1175-S1); all patients or their caretakers gave written consent. Samples were classified based on the final neuropathology report as either “schwannoma,” “meningioma,” or “other” tumors. The “other” category mostly included spinal metastases from various primary tumors, but also some rarer tumor entities such as spinal ependymomas or hemangioblastomas.

Samples were mounted on slides and imaged using the NIO Laser Imaging System (Invenio Imaging Inc., Santa Clara, CA, USA). Several regions of interest from the sample with varying sizes ranging from 1 by 1 to 8 by 8 mm in diameter, mostly 2 by 2 mm, were imaged and saved for further processing.

Samples were acquired from May 2021 to September 2024. An overview of all patients, their demographics and histopathological diagnoses is provided in (Supplement 1). Overall, the dataset consisted of 81 images from 22 patients with schwannomas, 56 images of 17 patients with meningiomas, and 95 images of 23 patients with other tumors, for a total of 232 images from 61 patients. The images of 20% of randomly selected patients were reserved for the test dataset used in the evaluation; out of the remaining patients, 20% were reserved for a validation dataset, with the images of the remaining patients being used for training the neural networks.

### Image Preprocessing

**Figure 1.**
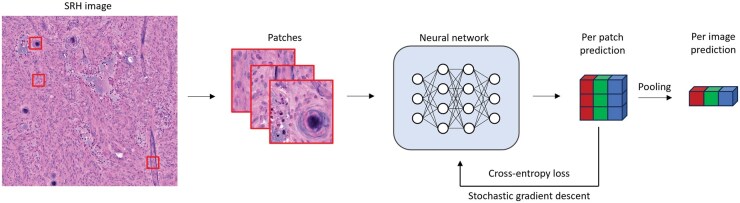
Graphical illustration of the processing pipeline. The reconstructed RGB image from the NIO imaging system was randomly sampled to create a total of 150 patches of size 224 by 224 pixels. These patches are then individually labelled with the histopathological tumor diagnosis (meningioma, schwannoma, or other spinal tumors) and used to train the neural network using cross-entropy loss. For test set inference, the patches are processed by the trained neural network and then aggregated using a pooling function (maximum or mean pooling).

The entire processing pipeline is illustrated in (Figure 1). Raw 2845 cm^–1^ (corresponding to the vibrational frequency of CH_2_ bonds) and 2940 cm^–1^ (corresponding to CH_3_ bonds) wavelength and 16-bit grayscale channels were converted to 8-bit RGB H&E-like images by the NIO imaging system. A total of 150 image patches with a size of 224 by 224 pixels each were then extracted from each H&E-like image at random locations for further processing by the neural network.

### Machine Learning Models and Training

We tested different computer vision neural network models for our study. To assess the performance of convolutional neural networks, we used the ResNet-50 model, which contains a total of 50 consecutive ResNet blocks. For Vision Transformers, we used the original model in its base configuration. In addition, we also tested the CTransPath architecture, a hybrid architecture combining elements of both convolutional neural networks and Vision Transformers, that was specifically designed for and trained on histopathological images.^15^ Models used either randomly initialized weights or weights pretrained on larger datasets. ResNet-50 and Vision Transformer models used weights pretrained on Imagenet; CTransPath was pretrained on images from the Cancer Genome Atlas (TCGA) and the Pathology AI Platform (PAIP).^16-18^

For model training, we labeled each patch created during image processing with the respective tumor class according to the histopathological diagnosis of the tissue as given by our neuropathology department. The models were trained for a total of 30 epochs using cross-entropy loss and ­stochastic gradient descent as the optimization algorithm; the model with the lowest loss on the validation dataset was used for evaluation with the test dataset.

### Model Testing

Model performance was based on the test dataset. For this evaluation, individual predictions (based on the softmax layer) for the patches extracted from each image were aggregated by either calculating the arithmetic mean of predictions (mean-pooling) or by choosing the patch with the most confident prediction as representative for the whole image (max-pooling), resulting in a classification for each image as either meningioma, schwannoma, or other tumors.

Statistical testing, including F_1_ score calculation and receiver operating characteristic (ROC) analysis, was performed using functions from the Scikit-learn Python module. Graphs were created with Matplotlib.

## Results

### Performance of Models and Pooling Strategies

Results for model accuracy and the respective F_1_ scores are shown in (Table 1). The highest diagnostic accuracy was achieved by combining a CTransPath model with max-pooling, achieving a diagnostic accuracy of 94.3%. The ResNet-50 model with mean-pooling achieved a total diagnostic accuracy of 88.8% across the whole test set. The Vision Transformer model achieved an overall accuracy above 90% regardless of the pooling function. The optimal pooling strategy based on our results is unclear, with max-pooling performing slightly better with the CTransPath model but significantly worse with the ResNet model.

**Table 1.** Table showing prediction accuracy (Acc.) on the test dataset for several different model architectures and pooling strategies, and for each tumor type separately, as well as the combined accuracy for all tumors and the F_1_ score

Model	Acc. Schwannomas	Acc. Meningiomas	Acc. Other	Acc. Overall	F_1_ score
CTransPath-MeanPool	77.8%	100%	87.5%	87.0%	0.869
CTransPath-MaxPool	100%	100%	87.5%	94.3%	0.945
ResNet-MeanPool	100%	91.6%	83.3%	88.8%	0.891
ResNet-MaxPool	100%	91.6%	66.7%	79.6%	0.789
ViT-MeanPool	94.4%	100%	87.5%	92.6%	0.926
ViT-MaxPool	100%	91.6%	83.3%	90.7%	0.909

### Prediction Probabilities, Confusion Matrix and ROC Analysis

**Figure 2.**
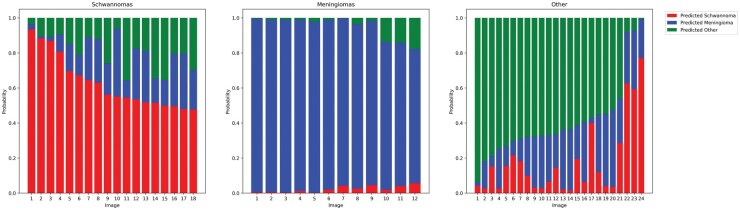
Overview of the prediction probability of the best-performing model. Every stacked bar corresponds to the prediction of a single image.

**Figure 3.**
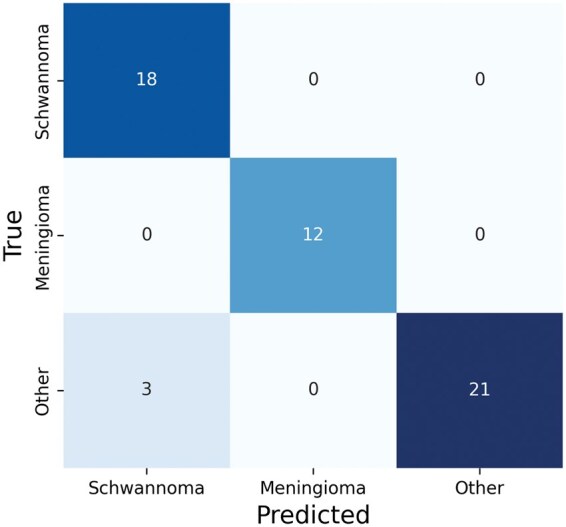
Confusion matrix of the best-performing model for the 3 available classes.

The prediction probabilities of the best-performing model, the max-pooled CTransPath model, are illustrated in (Figure 2). The model correctly identifies all meningiomas in the test dataset with high probability but is less confident in distinguishing schwannomas from other tumors, with 3 tumors misclassified as schwannomas (Figure 3).

**Figure 4.**
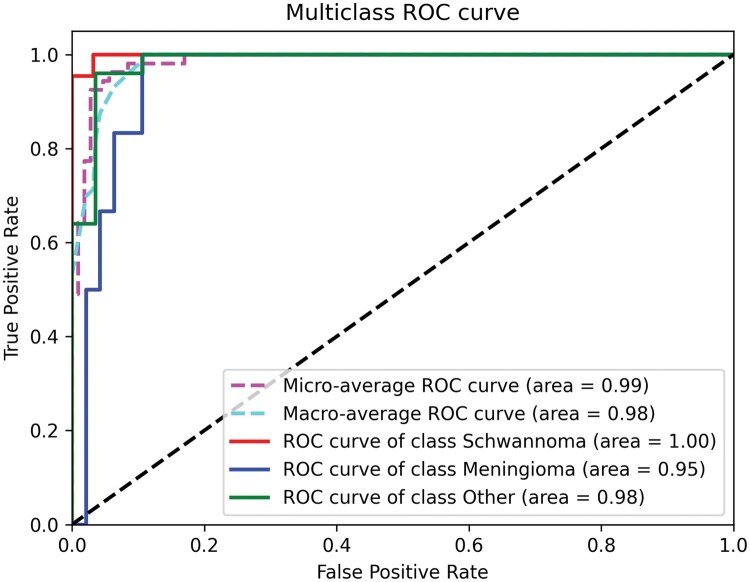
ROC analysis for the 3 classes separately as well as the micro- and macro-average ROC.

An ROC analysis of the best-performing CTransPath model is shown in (Figure 4). The model achieves very good performance for schwannomas and decent performance for meningiomas and other tumors, with an overall area of 0.99 for the micro-average and 0.98 for the macro-average ROC curves.

### Heatmaps of Model Inference

**Figure 5.**
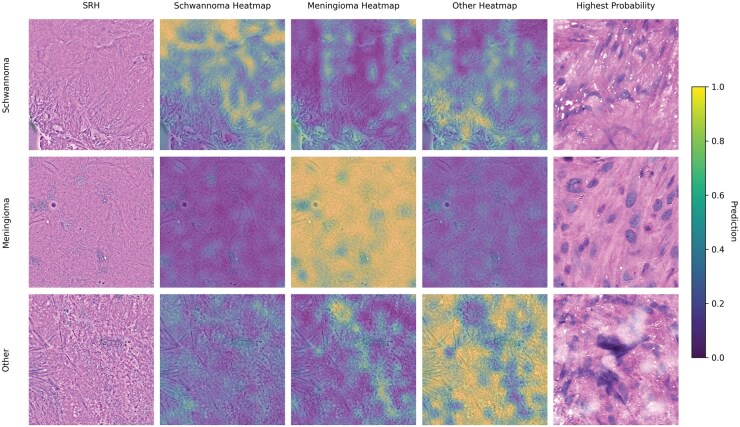
Heatmaps for prediction probabilities of individual patches for images from the 3 classes, using select SRH images shown on the left. The patches with the most confident correct prediction are shown on the right.

To illustrate how the models process information per patch, heatmaps for the results of model inference per patch for selected SRH images as well as the patch with the most confident correct prediction are shown in (Figure 5). For meningiomas, the model not only focuses on histological features such as psammoma bodies but can also reliably classify patches solely based on cellular patterns. For schwannoma, the model most confidently classifies cell-rich areas, ignoring larger histological features for example based on vasculature or fiber patterns.

### Effect of Pretraining on Model Performance

**Figure 6.**
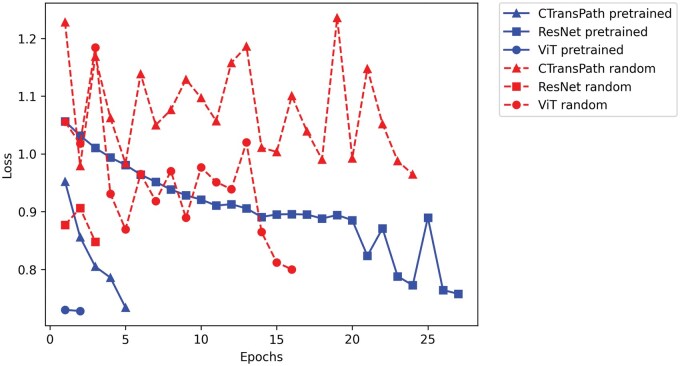
Validation cross-entropy loss for a ResNet-50, a base Vision Transformer, and a CTransPath model that were pretrained or randomly initialized. Losses are displayed up to the lowest overall validation loss that was achieved for each model.

To evaluate the effect of pretraining on model performance, we compared the validation losses of pre-trained Resnet-50 and CTransPath models to randomly initialized models across epochs; the results are illustrated in (Figure 6). The pretrained models successively improved across epochs, reaching their lowest validation loss after 2 epochs for the Vision Transformer, after 5 epochs for CTransPath and after 27 epochs for the ResNet architecture, respectively. Models without pretraining performed worse and did not converge as well, showing very high variability in validation loss across epochs.

## Discussion

To the best of our knowledge, our study is the first to use machine learning algorithms specifically for intraoperative histological differentiation of intradural extramedullary tumors.

Other tools that have been designed for rapid intraoperative tumor differentiation are, for example, based on regular Raman spectroscopy, as demonstrated by Klamminger et al.^19^ Similar to SRH, Raman spectroscopy can be used for rapid intraoperative tumor analysis. Unlike Raman histology, however, spectroscopy does not allow for actual histological imaging, resulting in classification performance inferior to both regular intraoperative histology and SRH. Fresh frozen sections are currently considered the gold standard for intraoperative spinal tumors. However, they require both skill for sample preparation and histological interpretation compared to the easily automated analysis of SRH.^20^ Intraoperative SRH is increasingly used for many different applications. AI-based analysis of SRH was shown to differentiate between tumor and non-tumor lesions and between different brain tumor entities. It can even detect molecular genetic features such as IDH mutations, 1p19q codeletions, and ATRX loss.^8^

Concerning our results, we achieved a diagnostic accuracy of 94.3% and a sensitivity of 100% for both meningiomas and schwannomas in our test set with our best model, making our intraoperative analysis competitive with histopathology based on fresh frozen sections, which reached a diagnostic accuracy of 96% in a study by Kobayashi et al.^20^ This is probably an overestimate for the actual diagnostic accuracy of our model on a prospectively collected dataset, as our test dataset may not be large enough to evaluate model performance accurately, and as our model was chosen from 6 different models and is thus slightly fitted to our test dataset. However, except for the ResNet-based model in combination with mean pooling, all of the models we tested achieved diagnostic accuracies above 85%, but with the advantage that histological analysis is automated, faster, and does not require intervention by a pathologist.^20^

Our best model was based on the CTransPath architecture. CTransPath was specifically designed to interpret histopathology and was also pretrained on histopathology images. The Vision Transformer and the max-pooled ResNet models performed only slightly worse, demonstrating that model architecture may not be that relevant as long as it is based on mechanisms suitable for image recognition, such as convolutional layers and attention.

Our model was trained and tested on a comparatively small dataset of only 61 patients. Although this is a decent number of cases given the rarity of the disease studied, this dataset would usually be far too small for adequate neural network training. However, several essential strategies have allowed us to achieve very high classification accuracy despite the small size of our dataset.

One strategy is using transfer learning to pre-train models on larger non-SRH image datasets such as ImageNet and TCGA. Pretraining allows the models to develop a general understanding of image classification before specializing in the actual task. Our study showed that pretraining leads to better performance on the validation dataset compared to a randomly initialized model.

The other important strategy is data augmentation, which is especially useful in histopathology. Histopathological whole slide images are generally far too large to be processed by a conventional computer vision model in one step. In addition, the image information contained within one slide is highly repetitive. As a result, most computer vision models working with histopathology images will only process smaller image patches instead of the whole slide. By processing 150 randomly chosen patches per image, we effectively increased the size of our dataset by the same factor.

In doing so, we effectively labelled and predicted every patch with the histopathological tumor diagnosis for supervised model training. This differs from the multiple instance learning-based approach that is well-established in the automated analysis of H&E histopathology. Campanella et al popularized these methods,^21^ showing promising results. Training techniques such as the one we used in our study showed poor results in H&E histopathology.

We believe that the discrepancy in performance for the methods we used is due to the unique characteristics of our SRH dataset. Our dataset is limited to CNS tumors. Unlike other tumor entities, CNS tumors are usually resected without a relevant margin to healthy tissue to prevent neurological deficits. As a result, our images contain very little non-tumor tissue, and simply labelling all randomly selected patches with the tumor diagnosis is a valid strategy compared to other organ systems, where healthy tissue may be misclassified. In addition, SRH imaging may be subject to fewer artefacts than H&E imaging, for example, due to sample staining or cutting.

One downside of our current analysis is that we focused on schwannoma and meningioma in our current study. Although there are other intradural extramedullary lesions, these two entities are by far the most frequent. Attempting to subclassify other tumors would have led to suboptimal results; however, we plan to create a more comprehensive spinal tumor classification model once we have a sufficiently large dataset. Concerning the quality of our AI-based analysis, many potential ways exist to improve classification. Current image analysis is based on the pooling of individual patch predictions. Whole slide image predictions based on techniques such as hierarchical transformers, graph neural networks, or multimodal large language models could further increase predictive power. However, these techniques are challenging to implement and may not be compatible with our current patch-based data augmentation strategy.

The rapid intraoperative histopathological analysis allows for effective intraoperative decision-making. In case of intradural extramedullary lesions, histopathological analysis would allow us to decide whether special care should be taken to identify any nerve roots that could be associated with the tumor. Other applications for intraoperative SRH include determining tumor margins, the extent of tumor resection, and identifying tumor subtypes in near-real-time to tailor the surgical approach accordingly. Intraoperative SRH could therefore improve surgical outcomes and patient prognosis.

In conclusion, our study demonstrates that AI-assisted analysis of SRH enables rapid and accurate intraoperative classification of intradural extramedullary tumors. Using a hybrid model architecture combining convolutional and transformer-based components, we achieved a diagnostic accuracy of 94.3%, highlighting its potential as a fast and reliable support tool for intraoperative histopathology. AI-interpreted SRH may enhance intraoperative decision-making, complement expert interpretation, and possibly contribute to improved surgical outcomes. Future work will aim to further increase diagnostic accuracy and extend applicability to a broader range of spinal tumor entities.

## Supplementary Material

vdaf211_Supplementary_Data

## Data Availability

The training, validation and test datasets are not publicly shared due to privacy concerns. Parts of the datasets consisting of data by patients that agreed to sharing the data with other researchers will be made available upon reasonable request to the corresponding author. The training code and the model weights for the best-performing model were made available on GitHub (https://github.com/pierrescheffler/idem).

## References

[1] Jenkinson MD , Simpson C, Nicholas RS, Miles J, Findlay GF, Pigott TJ. Outcome predictors and complications in the management of intradural spinal tumours. Eur Spine J. 2006;15:203-210. 16374649 10.1007/s00586-005-0902-x PMC3489409

[2] Ottenhausen M , Ntoulias G, Bodhinayake I, et al Intradural spinal tumors in adults—update on management and outcome. Neurosurg Rev. 2019;42:371-388. 29455369 10.1007/s10143-018-0957-x

[3] Koeller KK , Shih RY. Intradural extramedullary spinal neoplasms: radiologic-pathologic correlation. Radiographics. 2019;39:468-490. 30844353 10.1148/rg.2019180200

[4] Freudiger CW , Yang W, Holtom GR, Peyghambarian N, Xie XS, Kieu KQ. Stimulated raman scattering microscopy with a robust fibre laser source. Nat Photonics. 2014;8:153-159. 25313312 10.1038/nphoton.2013.360 PMC4193905

[5] Straehle J , Erny D, Neidert N, et al Neuropathological interpretation of stimulated raman histology images of brain and spine tumors: part B. Neurosurg Rev. 2022;45:1721-1729. 34890000 10.1007/s10143-021-01711-1 PMC8976804

[6] Neidert N , Straehle J, Erny D, et al Stimulated raman histology in the neurosurgical workflow of a major european neurosurgical center—part A. Neurosurg Rev. 2022;45:1731-1739. 34914024 10.1007/s10143-021-01712-0 PMC8976801

[7] Kondepudi A , Pekmezci M, Hou X, et al Foundation models for fast, label-free detection of glioma infiltration. Nature. 2025;637:439-445. 39537921 10.1038/s41586-024-08169-3 PMC11711092

[8] Hollon T , Jiang C, Chowdury A, et al Artificial-intelligence-based molecular classification of diffuse gliomas using rapid, label-free optical imaging. Nat Med. 2023;29:828-832. 36959422 10.1038/s41591-023-02252-4 PMC10445531

[9] He K , Zhang X, Ren S, Sun J. Deep residual learning for image recognition. In Proceedings of the IEEE Conference on Computer Vision and Pattern Recognition. 2016:770-778.

[10] Wang H , Qu T, Bernstein K, Barbee D, Kondziolka D. Automatic segmentation of vestibular schwannomas from T1-weighted MRI with a deep neural network. Radiat Oncol. 2023;18:78. 37158968 10.1186/s13014-023-02263-y PMC10169364

[11] Fuse Y , Takeuchi K, Hashimoto N, et al Deep learning based identification of pituitary adenoma on surgical endoscopic images: a pilot study. Neurosurg Rev. 2023;46:291. 37910280 10.1007/s10143-023-02196-w

[12] Vaswani A , Shazeer N, Parmar N, et al Attention is all you need. Adv Neural Inf Process Syst. 2017;30:5998-6008.

[13] Dosovitskiy A , Beyer L, Kolesnikov A, et alAn image is worth 16x16 words: Transformers for image recognition at scale. In International Conference on Learning Representations. 2021.

[14] Springenberg M , Frommholz A, Wenzel M, Weicken E, Ma J, Strodthoff N. From modern CNNs to vision transformers: assessing the performance, robustness, and classification strategies of deep learning models in histopathology. Med Image Anal. 2023;87:102809. 37201221 10.1016/j.media.2023.102809

[15] Wang X , Yang S, Zhang J, et al Transformer-based unsupervised contrastive learning for histopathological image classification. Med Image Anal. 2022;81:102559. 35952419 10.1016/j.media.2022.102559

[16] Deng J , Dong W, Socher R, Li L-J, Li K, Fei-Fei L. Imagenet: a large-scale hierarchical image database. In 2009 IEEE Conference on Computer Vision and Pattern Recognition. 2009:248-255.

[17] Weinstein JN , Collisson EA, Mills GB, et al; Cancer Genome Atlas Research Network. The cancer genome atlas pan-cancer analysis project. Nat Genet. 2013;45:1113-1120. 24071849 10.1038/ng.2764 PMC3919969

[18] Kang Y , Kim YJ, Park S, et al Development and operation of a digital platform for sharing pathology image data. BMC Med Inform Decis Mak. 2021;21:114-118. 33812383 10.1186/s12911-021-01466-1 PMC8019341

[19] Klamminger GG , Klein K, Mombaerts L, et al Differentiation of primary CNS lymphoma and glioblastoma using raman spectroscopy and machine learning algorithms. Free Neuropathol. 2021;2:26. 37284619 10.17879/freeneuropathology-2021-3458 PMC10240939

[20] Kobayashi K , Ando K, Ito K, et al Accuracy of intraoperative pathological diagnosis using frozen sections of spinal cord lesions. Clin Neurol Neurosurg. 2018;167:117-121. 29476934 10.1016/j.clineuro.2018.02.025

[21] Campanella G , Hanna MG, Geneslaw L, et al Clinical-grade computational pathology using weakly supervised deep learning on whole slide images. Nat Med. 2019;25:1301-1309. 31308507 10.1038/s41591-019-0508-1 PMC7418463

