# Supplementary material for: Artificial intelligence-based analysis and diagnosis of intradural extramedullary spinal tumors by stimulated Raman histology
Source: Neurooncol Adv. 2025 Oct 8;7(1):vdaf211. doi: 10.1093/noajnl/vdaf211 (PMC12746601; doi:10.1093/noajnl/vdaf211)
Supplement: vdaf211_Supplementary_Data [file vdaf211_supplementary_data.zip › Supplement 1.pdf]

Supplement 1: List of all included patients, demographics, datasets and histopathological diagnosis

Patient ID	Sex	Age	Number of Images	Set	Diagnosis
117	female	37	4	training	Schwannoma, CNS WHO grade 1
202	male	46	3	training	Schwannoma, CNS WHO grade 1
219	male	37	4	training	Schwannoma, CNS WHO grade 1
226	male	27	4	training	Cellular schwannoma, CNS WHO grade 1
416	male	51	2	training	Schwannoma, CNS WHO grade 1
458	male	30	3	training	Schwannoma, CNS WHO grade 1
536	male	64	4	training	Schwannoma, CNS WHO grade 1
538	male	49	2	training	Schwannoma, CNS WHO grade 1
577	female	69	3	training	Schwannoma, CNS WHO grade 1
585	female	47	2	training	Schwannoma, CNS WHO grade 1
610	male	56	3	training	Schwannoma, CNS WHO grade 1
686	male	59	3	training	Schwannoma, CNS WHO grade 1
934	female	71	4	training	Schwannoma, CNS WHO grade 1
300	male	36	3	validation	Schwannoma, CNS WHO grade 1
366	female	66	14	validation	Schwannoma, CNS WHO grade 1
883	male	39	2	validation	Schwannoma, CNS WHO grade 1
944	female	70	3	validation	Schwannoma, CNS WHO grade 1
515	male	70	4	test	Schwannoma, CNS WHO grade 1
527	male	38	4	test	Schwannoma, CNS WHO grade 1
559	female	61	7	test	Schwannoma, CNS WHO grade 1
666	female	39	3	test	Schwannoma, CNS WHO grade 1
11	female	78	4	training	Psammomatous meningioma, CNS WHO grade 1
231	male	37	5	training	Psammomatous meningioma, CNS WHO grade 1
310	female	82	4	training	Meningothelial meningioma, CNS WHO grade 1
452	female	83	3	training	Meningothelial meningioma, CNS WHO grade 1
478	female	86	1	training	Psammomatous meningioma, CNS WHO grade 1
489	female	85	4	training	Meningothelial meningioma, CNS WHO grade 1
523	female	50	2	training	Psammomatous meningioma, CNS WHO grade 1
708	male	66	2	training	Psammomatous meningioma, CNS WHO grade 1
727	female	65	2	training	Psammomatous meningioma, CNS WHO grade 1
832	female	66	3	training	Meningothelial meningioma, CNS WHO grade 1
839	male	71	2	training	Meningothelial meningioma, CNS WHO grade 1
931	female	61	3	training	Meningothelial meningioma, CNS WHO grade 1
986	female	70	3	training	Meningothelial meningioma, CNS WHO grade 1
555	female	54	6	validation	Meningothelial meningioma, CNS WHO grade 1
588	female	60	5	test	Psammomatous Meningioma, CNS WHO grade 1
604	male	66	4	test	Meningioma, CNS WHO grade 1
950	female	81	3	test	Meningothelial meningioma, CNS WHO grade 1
257	male	69	5	training	Metastasis of known renal cell carcinoma
306	male	72	3	training	Metastasis of a carcinoma
325	female	36	3	training	Localized, regressively altered tissue with predominantly cell- and capillary-rich granulation tissue and mixed-cell, round-cell infiltrates.
357	female	63	4	training	Plasmocytoma
374	female	77	4	training	Metastasis of known non-small cell carcinoma
436	male	84	3	training	Metastasis of a non-small cell carcinoma
512	female	40	3	training	Metastasis of a carcinoma
556	male	83	4	training	Metastasis of a melanoma
601	male	53	4	training	Metastasis of a non-small cell carcinoma, histomorphologically and immunohistochemically consistent with a metastasis of the known gastric carcinoma.
618	male	63	7	training	B-cell lymphoma
645	male	75	1	training	Collagenous connective tissue

704	male	80	2	training	Metastasis of a non-small cell carcinoma, histomorphologically and immunohistochemically consistent with a metastasis of a primary tumor from the lung.
936	male	36	3	training	Pilocytic astrocytoma, CNS WHO grade 1
273	male	19	12	validation	Capillary haemangioblastoma, CNS WHO grade 1
447	female	54	3	validation	Portions of connective tissue, cartilage, and fresh blood, without definitive evidence of viable tumor cells.
508	female	1	7	validation	Neuroblastoma
872	female	31	3	validation	Metastasis of a non-small cell carcinoma
176	female	65	5	test	Calcinosis surrounded by chronic granulomatous and resorptive inflammation in scarred striated muscle
234	male	9	4	test	Portions of necrotically degenerated tissue
267	male	80	4	test	Poorly differentiated metastasis of a non-small cell carcinoma
474	female	54	2	test	Metastasis of a non-small cell carcinoma, histomorphologically and immunohistochemically consistent with the presence of a metastasis of a breast carcinoma
939	female	80	6	test	Localized tissue with components of synovia and granulation tissue
989	female	75	3	test	Spinal ependymoma, CNS WHO grade 2
